# Evaluating the Use of Inhaled Budesonide and Ipratropium Bromide Combination in Patients at High Risk of Acute Respiratory Distress Syndrome Development: A Randomized Controlled Trial

**DOI:** 10.3390/ph18030412

**Published:** 2025-03-14

**Authors:** Hebatallah Ahmed Mohamed Moustafa, Faten H. Elbery, Ahmad Z. Al Meslamani, Sherouk M. Okda, Bshra A. Alsfouk, Amira B. Kassem

**Affiliations:** 1 Clinical Pharmacy and Pharmacy Practice Department, Faculty of Pharmacy, Badr University in Cairo, Cairo 11829, Egypt; 2 Department of Clinical Pharmacy, Faculty of Pharmacy, Al Salam University, Kafr Alzayat 31611, Algharbia, Egypt; pg_122660@pharm.tanta.edu.eg; 3 College of Pharmacy, Al Ain University, Abu Dhabi P.O. Box 112612, United Arab Emirates; ahmad.almeslamani@aau.ac.ae; 4 AAU Health and Biomedical Research Center, Al Ain University, Abu Dhabi P.O. Box 112612, United Arab Emirate; 5 Department of Clinical Pharmacy and Pharmacy Practice, Faculty of Pharmacy, Damanhour University, Damanhour 22514, Egypt; sherouk.okda@pharm.dmu.edu.eg; 6 Department of Pharmaceutical Sciences, College of Pharmacy, Princess Nourah bint Abdulrahman University, P.O. Box 84428, Riyadh 11671, Saudi Arabia; baalsfouk@pnu.edu.sa

**Keywords:** budesonide, ipratropium bromide, acute respiratory distress syndrome, inhalation therapy

## Abstract

**Objectives:** There is a scarcity of pharmacological treatments that efficiently address lung injury in individuals experiencing acute respiratory distress syndrome (ARDS). Early inhaled corticosteroids and ipratropium may reduce pulmonary inflammation and injury of the lungs, minimizing the risk of ARDS. **Method:** This is a double-blinded randomized control trial conducted on patients at risk of ARDS. Patients were randomly allocated into two groups; the intervention group (63 patients) were administered aerosolized budesonide and ipratropium bromide, and the control group (56) were administered a placebo every eight hours for five days. Alteration in oxygen saturation divided by inspired oxygen (Fio2) (S/F) after five days was the primary outcome. Secondary outcomes included ARDS occurrence, mechanical ventilation (MV) requirement, hospital stay duration, and mortality rates. **Results:** Of the 604 screened, only 119 patients were included. The intervention group (63 patients) S/F ratio recovered versus the fall of the control group. Both groups had similar organ dysfunction and 28-day mortality. The intervention group had significantly (*p* < 0.001) fewer cases developing ARDS (9.5%) and MV (9.5%) than the control group (46.4% and 35.7%, respectively). **Conclusions:** The administration of inhaled budesonide and ipratropium bromide improved oxygenation, as assessed by the S/F ratio, and significantly reduced the rate of ARDS development and the requirement of MV versus the control group. Larger multi-center trials including diverse patient populations are needed to validate these results.

## 1. Introduction

Although the diagnosis and management of acute respiratory distress syndrome (ARDS) have progressed significantly, ARDS still poses a significant risk of morbidity and mortality [1]. It is a complex heterogeneous syndrome with various clinical conditions or insults, such as pulmonary or non-pulmonary injury, infection, trauma, genetic susceptibility, or a cardiac event [2,3]. In ARDS, there is an exaggerated inflammation in lung tissue, degradation of the endothelium and epithelial pulmonary barriers, increased alveolocapillary permeability to proteins, inflammatory cells and platelet accumulation, microvascular coagulopathy, followed by non-cardiogenic lung edema, severe hypoxemia, acute respiratory failure, and an expansion of the lung’s dead space [3,4,5].

No one diagnostic test definitively confirms or rules out a diagnosis of ARDS [6]. The diagnosis of ARDS currently follows the 2012 Berlin criteria, which encompass clinical picture, arterial blood gas (ABG) analysis of pulmonary filtration ratio, chest imaging using X-rays showing bilateral lung opacities, higher ventilator settings in subjects who are mechanically ventilated, and multiorgan system failure [7,8,9]. Diagnoses of ARDS necessitate the presence of respiratory distress, either new or worsening, lasting for 7 days or less, radiographic infiltrates, and clinically substantial decreased oxygenation (Ferguson et al., 2012) [8]. Chest imaging reveals bilateral pulmonary radiographic abnormalities due to excessive alveolar capillary permeability, which is not cardiogenic and does not stem from volume overload or chronic pulmonary disease. Indeed, even quite straightforward clinical criteria fail to encompass the ARDS’s complexity and heterogenicity [6,10].

The Lung Injury Prediction Study (LIPS) developed the LIPS score to detect individuals at a high risk of developing ARDS upon emergency department (ED) admission. A LIPS score equal to or above 4 provides the optimum discriminating value, together with a positive predictive value of 18% [11]. A pulse oximeter reading of oxygen saturation (SpO2) divided by the Fio2 (S/F) can be used as the most reliable indicator of the development of ARDS, and it can be used instead of the PaO2/FiO2 (PF) ratio, which is calculated by utilizing the analysis of arterial blood gas (ABG) [12,13,14,15].

ARDS mortality most commonly results from refractory hypoxemia, sepsis, multiple organ failure, and respiratory failure [16,17]. Individuals who have survived ARDS are highly susceptible to experiencing muscle weakening, functional limitations, cognitive deterioration, and depression. Nevertheless, the majority of individuals regain normal or almost normal pulmonary function [18,19,20,21]. Across its severity spectra, ARDS is still underrecognized, especially in low-income countries with limited resources for chest imaging and ABG measurement, and is undertreated by lung-protective practices [22,23].

The management of ARDS is mainly supportive and it aims at limiting lung injury by timely management of the underlying insults to achieve a favorable prognosis [4,23]. Supportive interventions include lung-protective ventilator management [24], applying the prone position for 17 h per day or more [6,24], ventilator-acquired pneumonia (VAP) prevention and treatment [25], sensible fluid strategy to enhance pulmonary edema resorption [6,26], management of hypoxemia using supplemental oxygen, stress ulcer prophylaxis [6,27], using anticoagulants for prophylaxis against deep venous thrombosis [6], using neuromuscular blockers [28], sedation, and analgesia as needed for comfort provision [6], nutritional support (mainly enteral) [29], antibiotics for sepsis [23], restriction of blood products (to avoid volume overload and transfusion-associated lung injury) [23], and inhaled vasodilators such as nitric oxide [30]. Novel strategies for the management of ARDS include natural medicine therapy, cytokine-based therapy, cell-based therapy, and nanomedicine [31]. However, all the mentioned strategies cannot reverse the pathophysiological processes of ARDS [4].

Several pharmacologic therapies used in treating ARDS have failed to improve survival [4,32,33,34,35]. Although clear benefits were established with ventilation and fluid management of ARDS, decades of randomized controlled clinical trials of pharmacotherapies for ARDS showed conflicting results assessing drugs targeting inflammation, epithelial and endothelial injury, and coagulopathy [6,36]. Neutrophil elastase inhibition, statins, intravenous interferon-1a, surfactant replacement, anticoagulants, aspirin, ketoconazole, lysofylline, antioxidants, and vitamin D3 supplementation in deficient patients did not show any benefit in ARDS patients management [37,38,39,40,41].

Impaired fluid clearance from the alveoli in ARDS patients was associated with higher mortality rates [42]. When used for the management of patients with ARDS, β2-agonists such as salbutamol, in addition to being a bronchodilator, improved alveolar fluid clearance, preserved pulmonary vascular stability, and reduced extravascular lung water [43,44,45,46,47,48], even in individuals who did not have any injury to the epithelium of their alveoli before the occurrence of ARDS [49,50]. The administration of β2-agonists via inhalation in patients admitted for esophagectomy reduced pneumonia and other postoperative complications, but did not prevent ARDS [50]. Moreover, their use was associated with futility or harm [51,52]. Thus, the development of new, efficacious therapies for ARDS continues to be a challenge [26].

Corticosteroids can improve oxygenation and ameliorate histologic injury in numerous lung injuries of various origins that progress to ARDS. This benefit was especially demonstrated in patients admitted with pneumonia, where the use of systemic steroids reduced treatment failure [53,54,55]. Corticosteroids in prolonged low doses, such as methylprednisolone or dexamethasone, have been used under debate for inflammation management, enhancing ARDS resolution, and improving clinical outcomes [25,27,56,57].

Corticosteroids’ adverse effects include immunosuppression, critical illness neuropathy or myopathy, and adverse effects linked to delayed administration [36,57,58]. If a corticosteroid is to be used, it should be started before the 14th day of ARDS diagnosis [57,59]. Given that intravenous drugs can cause systemic side effects, the focus has initiated a shift toward enhancing local efficacy by delivering therapies directly to the respiratory tree and alveolar epithelium, while reducing systemic toxicities by using nebulization [4,26]. The involvement of inflammation in ARDS has led to the utilization of inhaled corticosteroids for its management and prevention [60,61]. Budesonide, an inhaled corticosteroid, exerts potent anti-inflammatory effects in the airways by binding to glucocorticoid receptors. It inhibits the production and release of cytokines, prostaglandins, and other inflammatory mediators, thereby reducing airway inflammation. Through these mechanisms, budesonide enhances airflow, reduces airway hyperresponsiveness, and improves oxygenation. Administering inhaled budesonide before lung ventilation improved cytokine profiles and lung compliance [62]. Possible preventative actions of inhaled corticosteroids and β2-agonists were suggested when utilized in animal studies or before hospitalization [63,64].

Ipratropium bromide is a short-acting anti-muscarinic agent with similar affinity to M1, M2, and M3 receptor subtypes. It alleviates bronchoconstriction by blocking muscarinic receptors in the bronchial smooth muscle, preventing acetylcholine-induced constriction. It is selectively distributed in the lungs, not well absorbed by the body when taken by mouth, and has fewer systemic adverse reactions when inhaled [65,66,67]. It is commonly administered by nebulization in mechanically ventilated patients. It can reduce sputum production, improve the pulmonary function of COPD patients, inhibit neutrophilic infiltration, and reduce pulmonary edema [68,69,70,71,72]. Ipratropium bromide requires 3–4 administrations daily [65]. Evaluating the efficacy of bronchodilator therapy should be based on several observations [73].

Reported adverse reactions with muscarinic antagonists include mouth dryness, urinary retention, taste disturbances, blurred vision, constipation, and, rarely, tachycardia [74]. It is contraindicated in people with prostatic hyperplasia, narrow-angle glaucoma, obstruction of the bladder’s neck, high-cardiovascular-risk patients, and those with serious heart rhythm abnormalities, myocardial infarction within the prior 6 months, and creatinine clearance of less than 50 mL/min for renally excreted anticholinergic agents [75]. Animal studies and in vitro studies documented that anti-muscarinic agents may have anti-inflammatory and anti-remodeling actions by acting on M3 receptors [76]. When used in premature calves with respiratory distress syndrome, a nebulized mixture of formoterol, ipratropium bromide, furosemide, and fluticasone propionate improved their lung function [77].

Following hospital admission, ARDS develops after about two days in susceptible individuals [11]. Currently, there is a fundamental shift toward ARDS prevention and early management of at-risk patients [10,23,78] via targeting epithelial and endothelial barrier repair, edema clearance, anti-inflammatory effect, and decreasing oxidative stress [26]. To our knowledge, the published research has not tested the protective effect of using inhaled corticosteroids and ipratropium bromide in subjects at high risk of ARDS development. Because drug repurposing and the use of bronchodilators may help in the prevention and early treatment of ARDS [26,49,73], this trial aimed to test the potential benefits of their administration to individuals at high risk of ARDS development (LIPS score higher than or equal to 4), added to the standard care, in oxygen saturation, ARDS occurrence, mechanical ventilation (MV) need, duration of hospital stay, and mortality rates.

## 2. Results

### 2.1. Study Details and Flowchart

**Figure 1:**
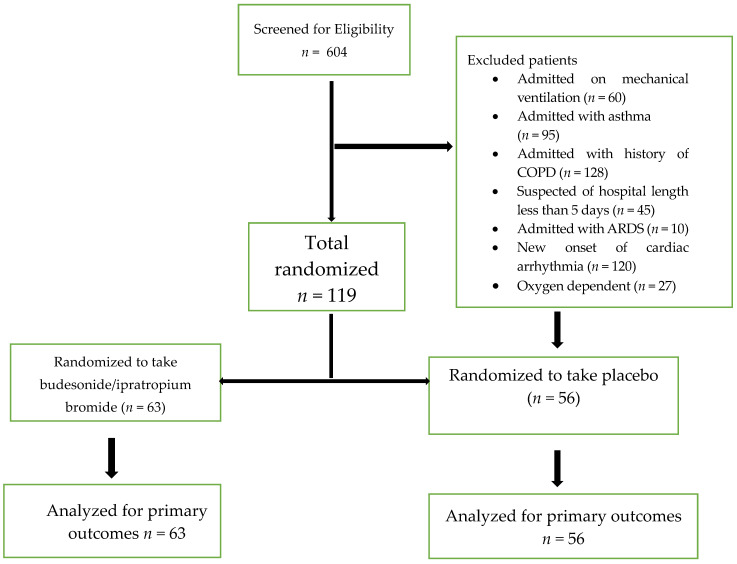
Study flow chart.

Figure 1 presents the Consolidated Standards of Reporting Trials diagram for this study. Our study included 119 patients in the analysis of the primary endpoint between November 2023 to April 2024. At first, 604 patients were screened for eligibility; 485 patients were excluded based on predefined criteria: 60 required mechanical ventilation, 95 had a diagnosis of asthma, 128 had a history of chronic obstructive pulmonary disease (COPD), 45 were expected to have a hospital stay of fewer than five days, 10 presented with acute respiratory distress syndrome, 120 had a new onset of cardiac arrhythmia, and 27 were oxygen-dependent. Consequently, 119 patients met the inclusion criteria and were randomized. After randomization, patients were allocated into the Budesonide/Ipratropium Bromide group (*n* = 63) and the placebo group (*n* = 56). No dropouts were reported, and all randomized participants completed the study protocol and were included in the final analysis for primary outcomes.

### 2.2. Patients’ Demographics, Risk Factors, and Comorbidities

The average age was similar between the control (64.6 years) and test (65.0 years) groups (*p* = 0.854). Significant differences were observed in sex distribution, with the test group having a higher percentage of females (74.6%) versus the control group (46.4%; *p* = 0.002) (Table 1). Hemorrhagic stroke was more prevalent in the control group (12.5%) compared with the test group (1.6%; *p* = 0.018). The test group had significantly higher incidences of pneumonia (65.1% vs. 25.0%; *p* = 0.001) and aspiration (57.1% vs. 21.4%; *p* = 0.001). Other notable differences included higher instances of hyperkalemia and hypoglycemia in the test group, though these did not reach statistical significance. The test group also had a higher prevalence of aspiration as a risk factor (57.1% vs. 21.4%; *p* = 0.001) and a lower ischemic heart disease comorbidity rate (9.5% vs. 23.2%; *p* = 0.042). The mean length of hospital stay did not significantly differ between both groups (control: 6.76 days, test: 6.75 days; *p* = 0.971).

### 2.3. S/F Ratio

**Figure 2:**
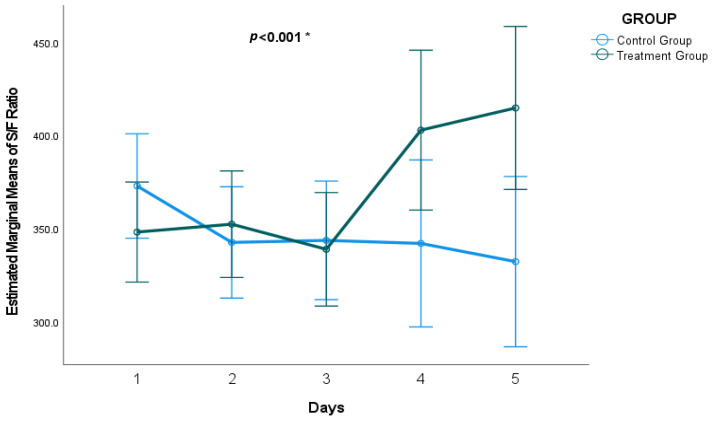
Oxygen saturation divided by FiO2 (S/F) ratio across treatment days. The plot displays the estimated marginal means of the S/F ratio through day 5, derived from mixed-effects modeling that incorporates study day, treatment, and their interaction (day × treatment). An asterisk (*) indicates days with a significant unadjusted *p*-value (<0.001) for the day × treatment interaction.

Table 2 summarizes the S/F ratio for the treatment and control groups over five days, along with the percentage change in the S/F ratio during this period. From day 1 to day 4, no significant differences in S/F ratio were observed between the treatment and control groups (*p* > 0.05). However, on day 5, the treatment group exhibited a significantly higher S/F ratio (401 ± 186.29) than the control group (316 ± 169.66, *p* = 0.011), corresponding to a moderate effect size (Cohen’s d = 0.58). Additionally, the total percentage change in the S/F ratio over five days was significantly greater in the intervention group (23%) compared with the placebo group (−11%) (*p* = 0.002), with an effect size (Cohen’s d = 0.62) indicating a moderate to large clinical impact. Figure 2 presents the estimated marginal means from our mixed-effects model.

### 2.4. SOFA Score

No significant difference in the severity of organ failure between the test and control groups was detected during the study period. Both groups had a median SOFA score of 2, indicating similar levels of organ dysfunction at the measured time point. The error bars, though very small, suggest minimal variability around the median score for both groups.

### 2.5. ABG Analysis

For normal ABG, the test group demonstrated a significant fluctuation from 33.9% on day 1 to 7.9% on day 2, then recovering to 31.7% by day 5, while the control group had more stable readings with a peak of 51.8% on day 4. A significant difference in the percentage of normal ABG between the treated and control groups was found (7.9% versus 28.6%, *p* = 0.003) on day 2 (Table 3). Respiratory acidosis was higher in the test group on most days, peaking at 38.1% on day 2, but, overall, did not show significant differences (*p* = 0.062). Metabolic acidosis was relatively stable in both groups without significant variation (*p* = 0.204). Respiratory alkalosis showed a notable drop in the test group from 28.6% on days 1 and 2 to 6.3% on day 4, while the control group remained higher on day 3 (33.9%; *p* = 0.036). Metabolic alkalosis occurrences were minimal and did not differ significantly between both groups (*p* = 0.394).

### 2.6. Biochemical and Physiological Parameters

Significant differences in median HCO3 between the test and control groups were noted on day 2 (23.0 versus 25.0, *p* = 0.011) and day 3 (20.7 versus 25.7 *p* = 0.002) (Table 4). The carbon dioxide (CO2) levels in the test group increased from 36.0 (28.0–43.0) on day 1 to 43.1 (33.5–45.0) on day 5 (*p* = 0.332), while the control group’s levels fluctuated less consistently (*p* = 0.147), with a notable difference on day 2 (*p* = 0.021). The pH values remained stable across both groups, showing no significant differences throughout the study period (test *p* = 0.962, control *p* = 0.934). The urea levels in the test group increased from 49.0 (42.0–170.0) on day 1 to 55.0 (50.0–218.0) on day 2, then decreased to 45.0 (44.0–145.0) by day 5 (*p* = 0.024). The control group’s urea levels showed a similar pattern, with significant changes observed (*p* = 0.014). The creatinine levels remained stable in both groups, with no significant differences observed (test: *p* = 0.147, control: *p* = 0.311). Oxygen saturation showed slight fluctuations without significant differences (test: 96.0 vs. control: 95.0, *p* = 0.127). Temperature remained stable across both groups (test: 37.8 vs. control: 37.8, *p* = 0.921). Heart rate showed significant differences, particularly on days 1 (95.0 vs. 85.0, *p* = 0.014), 2 (95.0 vs. 90.0, *p* = 0.032), and 3 (95.0 vs. 81.5, *p* = 0.041). Respiratory rate did not show significant differences between groups (test: 23.0 vs. control: 21.0, *p* = 0.224). WBC levels were significantly different on day 1 (8.6 vs. 12.9, *p* = 0.011), and overall differences were significant (test: 12.2 vs. control: 15.2, *p* = 0.002). Hemoglobin levels were stable with no significant differences (test: 10.9 vs. control: 10.9, *p* = 0.214). Platelet counts were similar across both groups (test: 255.0 vs. control: 255.0, *p* = 0.221). Sodium levels demonstrated no significant variation between both groups (test: 141.0 vs. control: 135.6, *p* = 0.205). Potassium levels were also similar (test: 4.1 vs. control: 4.2, *p* = 0.741). ALT levels were significantly different on day 1 (22.0 vs. 37.0, *p* = 0.021) and day 3 (16.0 vs. 27.0, *p* = 0.001). AST levels showed significant differences on day 2 (20.0 vs. 31.0, *p* = 0.021) and day 5 (22.0 vs. 29.0, *p* = 0.001). Glucose levels were significantly different on day 5 (207.0 vs. 187.0, *p* = 0.001). No drug-related adverse events occurred during the intervention.

### 2.7. Change in Mortality Rate and ARDS Development

The development of ARDS was significantly lower in the test group (9.5%) versus the control group (46.4%), with a *p*-value of 0.001 (Table 5). Additionally, the need for ventilation was significantly lower in the test group (9.5%) compared with the control group (35.7%), with a *p*-value of 0.001. The findings indicated that mortality rates did not differ significantly between the test and control groups over the 28 days (Table 5). On day 7, the test group reported a mortality rate of 4.8% (3 out of 63), compared with 7.1% (4 out of 56) in the control group, with a *p*-value of 0.582. By day 14, the mortality rates remained the same for both groups, with the test group at 4.8% and the placebo group at 7.1% (*p* = 0.582). By day 28, no further mortalities were reported in either group. The overall *p*-values for the total mortality rate was 0.212 for the test group and 0.122 for the control group.

### 2.8. Factors Influencing Percent Change in (S/F)

A linear regression model was developed to examine factors influencing the percentage change in the S/F ratio. The potential factors considered included the type of intervention (treatment vs. control group), patient age, gender, presence of pneumonia, aspiration at admission, risk factors, including aspiration, shock, or stroke, smoking status, history of past admissions, ABG measurements over five days, as well as SOFA and LIPS scores. Each factor was initially analyzed in relation to the S/F percentage change through univariate regression analysis. Subsequently, all interrelated factors were evaluated in a multivariate regression model. The results are shown in Table 6. The type of intervention was a significant predictor of S/F percent change in both univariate (B = 34.591, S.E. = 11.05, *p* = 0.002) and multivariate analysis (B = 51.612, S.E. = 12.780, *p* < 0.001). Moreover, SOFA score (B = 11.430, S.E. = 4.993, *p* = 0.024), and ABG on day 3 (B = −33.223, S.E. = 15.804, *p* = 0.038) were identified as significant factors influencing the percentage change in the S/F ratio in the multivariate analysis. Aspiration at admission was significant in univariate analysis (*p* = 0.009), but not in the multivariate model.

### 2.9. Factors Associated with the Development of ARDS

The logistic regression analysis presented in Table 7 evaluates factors associated with the development of ARDS. The treatment group compared with the control group was linked to decreased odds of developing ARDS (B = −2.801, OR = 0.061, *p* < 0.001), while other parameters, including age, gender, septic shock, pneumonia, aspiration, shock, stroke, past admission within the last 90 days, LIPS score, and arterial blood gas (ABG) values over five days did not exhibit statistical significance (*p* > 0.05).

### 2.10. Factors Associated with the Need for MV

Another logistic regression model was conducted to test the association between the need for MV and factors potentially contributing to it, such as the type of intervention, age, gender, S/F ratio at day 5, S/F percentage change over five days, septic shock, pneumonia, aspiration, shock, stroke, past admission within the last 90 days, LIPS score, and ABG over five days (Table 8). The treatment group was the only factor significantly associated with a reduced need for MV (B = −1.960, OR = 0.141, *p* = 0.01).

### 2.11. Mixed-Effects and Multilevel Regression Analysis of Treatment Outcomes

A multilevel regression analysis was conducted to examine the effect of the treatment group on the S/F ratio, controlling for time, pulmonary edema, pneumonia, and their interactions. The results indicated a significant effect of the treatment group on the S/F ratio (F = 18.125, *p* < 0.001). Additionally, there were significant interactions between the treatment group and both pulmonary edema (F = 7.071, *p* < 0.001) and pneumonia (F = 17.660, *p* < 0.001), suggesting that the treatment effect varied depending on the presence of these conditions. When stratified by pulmonary edema status, the treatment effect remained significant in patients without pulmonary edema (F = 19.643, *p* < 0.001), but was not significant in those with pulmonary edema (F = 0.072, *p* = 0.792). Conversely, in the pneumonia-stratified analysis, the treatment effect remained significant in both patients with pneumonia (F = 12.395, *p* < 0.001) and non-pneumonia patients (F = 4.569, *p* = 0.036).

Furthermore, a significant interaction between time and treatment group was observed (F = 2.905, *p* = 0.023), indicating that the treatment effect varied over time. However, time alone was not a significant predictor of the S/F ratio (F = 1.751, *p* = 0.141). The fixed effects results are presented in Table 9. Additionally, Table 9 reports variance estimates for repeated measures and random effects, highlighting the contribution of gender, aspiration, shock, IHD, LIPS, and SOFA score to variability in the S/F ratio. The repeated measures variances increased over time, with higher variability at later time points.

## 3. Discussion

ARDS is characterized by pulmonary inflammation and damage of the pulmonary barriers, with pulmonary and non-pulmonary complications [3,4,5]. In this randomized controlled clinical trial, the administration of inhaled budesonide and ipratropium bromide to subjects who were at high risk of ARDS was safe and effective at improving oxygenation. This was shown by the longitudinal improvement in the S/F ratio and the significantly lower rates of ARDS development and MV requirement.

Over time, the test group experienced an increase in the S/F ratio, while the control group exhibited a slight decrease by the end of the five days. The S/F ratio was previously suggested as a reliable surrogate outcome and an ideal predictor of ARDS progression and death for patients at risk of ARDS [14]. It correlated well with the Pao2/Fio2 ratio [8,79]. Hypoxemia is an independent risk factor linked to higher rates of morbidity and mortality [12]. In addition, a previous study found that mortality in patients who had sepsis and ARDS was mainly correlated with the severity of hypoxemia [80]. In a previous randomized trial, SpO2/FiO2 levels improved over time in people with a high risk of ARDS who were administered inhaled corticosteroids and β2-agonists [49]. Nebulized budesonide improves oxygenation and decreases inflammatory markers without changing hemodynamics [81].

The 28-day mortality rate was not significantly different between the two groups. Similarly, the combination of β2-agonists and corticosteroids did not reduce mortality, although it prevented ARDS development [82]. Researchers linked the use of inhaled steroids before hospitalization to a reduced risk of developing ARDS [83]. Moreover, corticosteroids may improve oxygenation, decrease inflammation, and hasten radiographic improvement without adding survival benefits [57]. The use of dexamethasone in subjects who already developed ARDS may decrease the use of MV and decrease the 60-day mortality [58]. Notably, its use in subjects with COVID-19 improved their survival [84].

Effective therapies that decrease mortality rates in established ARDS are still needed. According to animal studies, pharmacological drugs that prevent lung injury failed to establish real benefits after lung injury development [82]. In addition, although inhaled nitric oxide improved oxygenation in clinical trials and improved long-term pulmonary function, it did not decrease mortality rates and was associated with acute renal failure [85,86]. Similarly, the use of a high dose of ascorbic acid infusion failed to reduce 28-day mortality in individuals with transfusion-related acute lung injury (TRALI), although it was associated with less hypoxia [87]. However, another trial reported that a high dose of ascorbic acid in individuals with early sepsis and ARDS did not affect the organ failure assessment score at 96 h, but significantly reduced the 28-day all-cause mortality [88].

Although smokers are at an increased risk of ARDS occurrence [89], smoking history did not differ significantly between both groups, which may have impacted clinically unapparent airway responsiveness. No statistically significant difference was reported between both groups at baseline concerning the rate of sepsis or septic shock. The presence of sepsis is linked to a more severe illness, worse outcomes, and increased mortality rates [90,91,92].

We found a statistically significant difference between both groups at baseline concerning the rates of pneumonia, hemorrhagic stroke, and aspiration. Both pneumonia and aspiration are risk factors for ARDS [93,94]. Aspiration of oropharyngeal or gastric contents is a direct insult to the pulmonary system and is a recognized cause of ARDS. Stroke patients, due to dysphagia and impaired protective reflexes, are particularly susceptible to aspiration events. The altered level of consciousness in hemorrhagic stroke patients predisposes them to aspiration events, thereby elevating the risk of ARDS [94]. The introduction of foreign material into the lungs can cause severe inflammation and damage to the alveolar–capillary membrane, precipitating ARDS. Hemorrhagic stroke was significantly higher in the control group at baseline. It was previously reported that ARDS development risk was higher with direct (pulmonary) than indirect (non-pulmonary) injury [95]. However, although the test group had significantly higher percentages of pneumonia and aspiration at baseline, the rate of ARDS development was lower in the therapy group than in the placebo one. Patients with acute hypoxemia and pneumonia may have better responses to treatment [96]. Systemic corticosteroid use led to better treatment outcomes in ARDS patients with pneumonia in previous trials [54,55].

The percentage of patients with ischemic heart disease was significantly higher at baseline in the control group. While ischemic heart disease itself is not a direct risk factor for ARDS, it can exacerbate the condition’s severity. The presence of ischemic heart disease can worsen gas exchange abnormalities and increase the risk of ARDS development [97]. Regarding the results of linear regression analysis, the treatment group showed a significant effect on S/F ratio percentage change in both univariate and multivariate models. This suggests that the treatment contributed to an improvement in the S/F ratio, even when accounting for baseline differences in factors such as hemorrhagic stroke, pneumonia, aspiration at admission, and aspiration as a risk factor, which differed between the treatment and placebo groups. Importantly, the effect of the treatment remained consistent despite these confounding factors. These findings were further supported by the logistic regression analysis, where the treatment group was associated with a lower likelihood of developing ARDS and a reduced need for MV, even after adjusting for other relevant clinical parameters.

The test group had lower requirements for MV. MV is essential to manage individuals with ARDS [12]. However, there was no significant difference between both groups concerning the duration of hospital stay or mortality rates. A previous systematic review on the use of steroids in ARDS reported that they reduced the use of MV in ARDS. However, they were unable to decrease death rates or result in better clinical outcomes [36]. On the other hand, corticosteroid treatment decreased the MV need, increased ICU-free days, and reduced mortality risk without increasing the risk of nosocomial infection, according to a previous meta-analysis [25].

The treated subjects in the current study had lesser rates of ARDS development. Two previous studies reported that the utilization of inhaled corticosteroids and/or β2-agonists before hospitalization may protect against the progression to ARDS, especially in those with pneumonia [63,64]. The administration of inhaled corticosteroids to treat lung injury either alone or combined with N-acetylcysteine or β2-agonists in animal models has demonstrated repair of histologic injury and improvement of hypoxia [60,61,98,99]. Moreover, systemic administration of corticosteroids improved treatment outcomes in ARDS patients with pneumonia [54,55,100].

The results of the multilevel regression analysis highlight the significant effect of the treatment group on the S/F ratio, suggesting a beneficial impact of the intervention. However, the observed interactions between treatment and both pulmonary edema and pneumonia indicate that the effectiveness of treatment varies depending on these conditions. Notably, in the pulmonary edema-stratified analysis, the treatment effect remained significant in patients without pulmonary edema, but was not significant in those with pulmonary edema. This finding suggests that pulmonary edema may attenuate the response to treatment, possibly due to fluid accumulation impairing lung compliance and gas exchange. In contrast, in the pneumonia-stratified analysis, the treatment effect remained significant in both patients with pneumonia and non-pneumonia patients, though with a slightly lower significance level in the latter group. This suggests that the intervention maintains effectiveness, regardless of pneumonia status, though the difference in statistical strength may indicate varying degrees of responsiveness.

The observed interaction between time and the treatment group further emphasizes that the treatment effects evolved. This may reflect dynamic physiological changes during recovery or disease progression, underscoring the need for continuous monitoring. However, time alone was not a significant predictor of the S/F ratio, suggesting that treatment effects are not solely driven by the passage of time, but rather by the intervention itself and patient-specific factors. Increasing variability in repeated measures suggests heterogeneous patient responses, likely influenced by disease progression and individual characteristics such as gender, aspiration, shock, IHD, LIPS, and SOFA score. Overall, these findings underscore the importance of considering individual patient characteristics, particularly pulmonary edema status, when evaluating treatment efficacy. Future research is warranted to further investigate the underlying mechanisms driving these variations and to identify strategies for optimizing treatment efficacy across different patient subgroups.

### Strengths and Limitations

This clinical trial is the first one to report the efficacy and safety of inhaled budesonide and ipratropium administration in individuals with increased risk of ARDS development. In addition, their affordability and wide availability make them potential therapies for ARDS prevention and treatment. Moreover, the enrollment of subjects with high risk of ARDS at the ED was feasible, in line with a previous study [49]. However, our study has some limitations. First, there were some imbalances between groups at baseline, similar to a previous study [49], which may affect the interpretation of this study’s findings. Second, the S/F ratio is susceptible to measurement variations due to factors like pulse oximetry inaccuracies and timing of assessment. Additionally, confounding variables, such as differences in clinical management, might have influenced both the S/F ratio changes and the subsequent decision to initiate mechanical ventilation. Furthermore, the heterogeneity of the patient population may limit the generalizability of our findings. However, regression models controlled for potential confounders, strengthening the validity of treatment effects. Second, the generalizability of our results might be limited by the relatively small sample size. However, our sample size was much higher than that of a similar trial [49]. Third, biomarkers of inflammation or lung injury were not measured, which could have provided additional mechanistic insights into the effects of the intervention. Future studies incorporating inflammatory biomarkers could help in further elucidating the biological impact of the treatment. Finally, our study did not include long-term follow-up data, which could provide insights into the sustained benefits and potential risks of prolonged inhaled corticosteroid use. Future research with standardized measurement protocols, carefully controlled intervention, extended follow-up, and diverse patient populations is warranted to assess long-term outcomes and optimize treatment strategies. Future studies should explore how factors like sepsis, aspiration, and pulmonary edema influence treatment response.

## 4. Patients and Methods

### 4.1. Study Design

A double-blinded, randomized controlled clinical trial (Supplementary File).

### 4.2. Setting

ICU department (20 beds) in Mashtoul El Souq Governmental Hospital (50 beds), Sharkia, Egypt. We enrolled 119 patients from November 2023 to April 2024. The trial was registered on clinicaltrials.gov with the ID NCT06657079 (https://clinicaltrials.gov/study/NCT06657079, accessed on 1 January 2025).

### 4.3. Inclusion Criteria

Patients were 18 years or older, presented to the emergency room, and had one known risk factor for ARDS or more, such as aspiration, acute abdomen, shock, and serum albumin less than 3. They also had to have a LIPS score of 4 or higher and acute hypoxemia, which was defined as needing 2 liters per minute of extra oxygen or more to keep their oxygen saturation level in the range of 92–98% [49].

### 4.4. Exclusion Criteria

Pregnant patients; those who are not able to provide consent within twelve hours of admission; indications or contraindications for either corticosteroids or ipratropium (allergy to either budesonide and/or ipratropium bromide use, history of asthma or chronic obstructive lung disease, acute coronary disease, new onset arrhythmia, high cardiovascular risk patients, those with serious heart rhythm abnormalities, myocardial infarction within the prior 6 months, uncontrolled atrial fibrillation, or persistent sinus tachycardia of >130/minute after treatment with fluids, vasopressors, antimicrobials, and oxygen), administration of inhaled muscarinic antagonist or glucocorticoids in the previous week, systemic glucocorticoid treatment on admission or up to seven days before admission ≥ 5 mg of prednisone each day, occurrence of ARDS (diagnosed according Berlin criteria) before enrollment [8], acute lung injury prior to randomization, prostatic hyperplasia, narrow-angle glaucoma, obstruction of the bladder’s neck, undergoing mechanical ventilation before admission (ventilator dependent subjects), heart failure presentation without risk factors for ARDS, expected in-hospital stay, and/or survival less than two days or admission to receive hospice care only, or patient, surrogate or physician not committed to full support (except for an individual treated with all supportive care except resuscitation after cardiac arrest will not be excluded) [49].

### 4.5. Randomization and Intervention

Within 12 h of the ED’s presentation, a randomization tool [101] assigned patients in a 1:1 allocation ratio. The intervention group (63 patients) received budesonide administered at 0.5 mg/2 mL every 12 h and ipratropium bromide (500/2 mL) aerosolized every eight hours for up to five days. The intervention group administered budesonide and ipratropium bromide using jet nebulizers with an aerosol particle size of less than 5.5 µm. The first dose of the drugs was delivered up to four hours post-randomization (with a maximum delay of 16 h from ED presentation).

A placebo (normal saline) was used in the control group (56 patients) in place of ipratropium bromide, carefully matching the active drug in appearance to prevent participants from distinguishing between them, in addition to standard clinical care. Additionally, a sterile 0.9% NaCl inhalation solution was utilized as a placebo for budesonide, replicating its physical properties without containing the active ingredient. The placebo group received similar-looking solutions, and the medication was administered by a blinded respiratory therapist up to 4 h post-randomization.

To maintain blinding, identical packaging and labeling were used for both active treatments and placebos, ensuring that neither participants nor study personnel could differentiate between them. The principal investigator (PI) implemented a coding system, assigning unique identifiers to treatments instead of using drug names. The PI was solely responsible for unblinding the treatment assignments, which occurred only at the end of the trial for statistical analysis. No emergencies that required unblinding occurred during this study.

### 4.6. Measurements

At baseline, we reported the baseline characteristics of the patients, such as demographics, smoking status, medical conditions on admission, and risk factors. Medical conditions requiring past admissions and the length of stay was also recorded. After obtaining consent, the baseline S/F was evaluated using an air entrapment mask (oxygen saturation titrated to 94% ± 2% unless the goal was met on room air or the clinical situation required ventilation) every day for up to five days by a respiratory therapist before the morning dose. In addition, the daily changes in arterial blood gas parameters and the daily variations in the biochemical and physiological parameters were reported for both groups.

### 4.7. Outcomes

Our primary outcome was the alteration in S/F over up to five days. The SF ratio can replace PaO2/FiO2 (P/F) as a noninvasive surrogate that detects individuals with ARDS using the non-invasive pulse oximetry method [13]. The secondary outcomes were the occurrence of ARDS, which was determined using Berlin criteria after a chest radiograph [8], the length of stay, the need for MV, and the rates of all-cause mortality.

### 4.8. Statistical Analysis

The intention-to-treat analysis principle was used. We used SPSS, version 26, to analyze the data. Continuous variables were assessed for normality of distribution using the Kolmogorov–Smirnov test, and the *p*-value was found to be 0.091, which suggests that these variables are normally distributed. Hence, we used the mean and standard deviation (SD) to present them. Cochran’s Q Test was used to test for differences in proportions of blood gas parameters over time points. The Mann–Whitney U test and Friedman test were used to test for differences in medians, and an independent Student’s t-test was used to test for differences in means as appropriate. Multiple linear regression analysis was conducted to detect potential factors influencing the percentage change in the S/F ratio. Additionally, logistic regression was conducted to identify factors contributing to the development of ARDS and factors associated with the requirement of MV. Microsoft Excel and GraphPad Prism (https://www.graphpad.com/features, accessed on 6 December 2024) were used to create artwork. A *p*-value of less than 0.05 was considered significant.

Because an initial inspection (histograms and normality tests) indicated that S/F ratio data were not strictly normally distributed, we present raw data on each day as the median and interquartile range (IQR) in Table 2 and use the Mann–Whitney U test for simple unadjusted comparisons between groups. However, to properly account for repeated measures (day 1 to day 5 within each patient) and to evaluate the overall effect of treatment over time, we also fit a mixed-effects (multilevel) model. This model included fixed effects for treatment, day, and treatment × day interaction, as well as random intercepts for patients to address the within-subject correlation. From this model, we obtained estimated marginal means of the S/F ratio at each time point by group. These marginal means (presented in Section 2.3 and Figure 2) reflect an adjusted summary of the data and allow us to test the treatment effect across the study period, specifically via the day × treatment interaction term.

The final interpretation of treatment effectiveness is based primarily on this repeated-measures approach, as it captures changes over time and controls for individual-level variability. Due to observed imbalances in certain baseline characteristics, we performed multivariable regression to adjust for covariates in our analysis of ARDS development and mechanical ventilation. Specifically, we included [list key covariates, e.g., pneumonia, aspiration, stroke type, etc.] in the model based on clinical relevance and univariate screening. Additionally, we conducted multilevel regression to account for hierarchical data structures and variability across patient groups. For the longitudinal analysis of the S/F ratio, we utilized a mixed-effects (multilevel) model with a random intercept for each patient, accounting for repeated measures across days 1 to 5 and potential confounders.

### 4.9. Sample Size

According to a previous study, this intervention led to an approximately 60% decrease in ARDS [49]. Based on calculations performed using the G*power software (Accessed on 2 May 2023) [102], and considering a 5% level of significance, 0.8 statistical power, and an 8% attrition rate, the considered sample size for each arm was 57.

## 5. Conclusions

In patients at high risk of developing ARDS, early combined use of inhaled budesonide and ipratropium significantly increased oxygenation, decreased MV need, and was linked to lower rates of ARDS development. However, it did not significantly impact mortality or hospital stay duration. These findings highlight the potential of this therapeutic approach in preventing ARDS and improving patient outcomes. This study’s results should be validated using multi-center larger clinical trials to establish its efficacy and safety in broader patient populations. Future studies should explore how factors like sepsis, aspiration, and pulmonary edema influence treatment response. This study might be interesting to ARDS researchers and may help to guide future clinical trials testing the repurposing of pharmacological therapies for ARDS prevention and exploring the biological pathways by which they modulate lung injury and inflammation.

## Figures and Tables

**Table 1:** Baseline characteristics of patients.

Characteristic	Test (*n* = 63)	Control (*n* = 56)	*p*-Value
Age, mean (SD)	65.0 (10.2)	64.6 (12.2)	0.854
Sex			
Female	47 (74.6%)	26 (46.4%)	0.002 *
Male	16 (25.4%)	30 (53.6%)	
Smoking Status			
Smoker	4 (6.3%)	3 (3.3%)	0.818
Non-smoker	59 (93.7%)	53 (94.6%)	
Medical condition on admission **			
Ischemic Stroke	29 (46.1%)	34 (54.0%)	0.349
Hemorrhagic Stroke	1 (1.6%)	7 (12.5%)	0.018 *
Post Ictal	1 (1.6%)	0 (0.0%)	0.344
ACS	2 (3.6%)	3 (4.8%)	0.946
AF	0 (0.0%)	1 (1.8%)	0.287
Hypertension	5 (7.9%)	2 (3.6%)	0.201
Pulmonary Edema	8 (12.7%)	4 (7.1%)	0.205
Cardiogenic Pulmonary Edema	7 (11.1%)	2 (3.6%)	0.104
Pneumonia	41 (65.1%)	14 (25.0%)	0.001 *
Aspiration	36 (57.1%)	12 (21.4%)	0.001 *
Septic Shock	4 (6.3%)	0 (0.0%)	0.055
Sepsis	12 (19.0%)	9 (16.1%)	0.671
Hepatic Encephalopathy	1 (1.6%)	2 (3.6%)	0.614
UTI	6 (9.5%)	3 (5.4%)	0.391
AKI	27 (42.9%)	17 (30.4%)	0.214
Severe Hypokalemia	3 (4.8%)	3 (5.4%)	0.882
Hyperkalemia	4 (6.3%)	0 (0.0%)	0.055
Hyperglycemia	0 (0.0%)	1 (1.8%)	0.287
Hypoglycemia	4 (6.3%)	0 (0.0%)	0.055
Severe Anemia	2 (3.2%)	6 (10.7%)	0.187
Unstable Angina	1 (1.6%)	2 (3.6%)	0.491
Risk Factors			
Aspiration	36 (57.1%)	12 (21.4%)	0.001 *
Acute Abdomen	0 (0.0%)	3 (5.4%)	0.063
Shock	9 (14.3%)	5 (8.9%)	0.365
Albumin Less Than 3	6 (9.5%)	11 (19.6%)	0.115
LIPS, mean (SD)	5.7 (1.4)	5.6 (1.6)	0.591
Medical conditions occurred past admissions			
Hypertension	44 (69.8%)	39 (72.2%)	0.981
Atrial fibrillation	3 (4.8%)	5 (9.3%)	0.365
Strokes	13 (20.6%)	10 (17.9%)	0.702
Heart failure	1 (1.6%)	2 (3.6%)	0.491
Ischemic Heart disease	6 (9.5%)	13 (23.2%)	0.042 *
Cardiomyopathy	0 (0.0%)	2 (3.6%)	0.13
Diabetes	23 (36.5%)	26 (46.4%)	0.272
Chronic kidney disease	12 (19.0%)	8 (14.3%)	0.488
Length of stay, mean (SD)	6.75 (1.5)	6.76 (1.9)	0.971

* Statistically significant result *p* < 0.05. ** % within test or control. ACS: Acute coronary syndrome, AF: atrial fibrillation, UTI: urinary tract infection, AKI: acute kidney injury, LIPS: Lung Injury Prediction Score. Results are represented as mean ± SD or number (%).

**Table 2:** Comparison of (S/F) ratio and percentage change between treatment and control groups.

Parameter	Treatment Group *n* = 63	Control Group *n* = 56	*p*-Value
(S/F) day 1	438 (214/447)	438 (300/447)	0.710
(S/F) day 2	438 (210/460)	316 (258/450)	0.401
(S/F) day 3	334 (269/447)	442 (206/452)	0.321
(S/F) day 4	442 (264/447)	442 (188/457)	0.566
(S/F) day 5	438 (255/452)	438 (100/452)	0.263
(S/F) median percent change	1.53 (−2/41)	0 (−54/17)	0.037 *

A total percentage change in (S/F) over five days. S/F Ratio: peripheral oxygen saturation (SpO_2_)/fraction of inspired oxygen (FiO_2_) Ratio. Results presented as the median (25th percentile/75th percentile). * Statistically significant result *p* < 0.05. Mann–Whitney test was used to compare treatment and control groups.

**Table 3:** Daily changes in arterial blood gas parameters in test versus control arms.

Parameter	Arm	Day 1 (Baseline)	Day 2	Day 3	Day 4	Day 5	*p* Value (Total)
Normal ABG	Test	19 (33.9%)	5 (7.9%)	17 (30.4%)	23 (36.5%)	20 (31.7%)	0.142
	Control	19 (30.2%)	16 (28.6%)	13 (20.6%)	29 (51.8%)	23 (41.1%)	
	*p* value (subtotal)	0.660	0.003	0.223	0.094	0.291	
Respiratory Acidosis	Test	17 (27.0%)	24 (38.1%)	12 (19.0%)	16 (25.4%)	16 (25.4%)	0.062
	Control	13 (23.2%)	16 (28.6%)	8 (14.3%)	7 (12.5%)	13 (23.2%)	
	*p* value (subtotal)	0.255	0.272	0.488	0.075	0.782	
Metabolic Acidosis	Test	11 (17.5%)	12 (19.0%)	10 (15.9%)	7 (11.1%)	7 (11.1%)	0.204
	Control	6 (10.7%)	8 (14.3%)	5 (8.9%)	6 (14.3%)	2 (3.6%)	
	*p* value (subtotal)	0.294	0.488	0.309	0.567	0.177	
Respiratory Alkalosis	Test	18 (28.6%)	18 (28.6%)	10 (15.9%)	4 (6.3%)	13 (20.6%)	0.160
	Control	17 (30.4%)	18 (32.1%)	19 (33.9%)	7 (12.5%)	12 (21.4%)	
	*p* value (subtotal)	0.831	0.672	0.036	0.338	0.560	
Metabolic Alkalosis	Test	1 (1.6%)	4 (6.3%)	1 (1.6%)	3 (4.8%)	1 (1.6%)	0.394
	Control	1 (1.8%)	2 (3.6%)	1 (1.8%)	0 (0.0%)	0 (0.0%)	
	*p* value (subtotal)	0.933	0.489	0.564	0.459	0.287	

ABG: Arterial blood gas.

**Table 4:** Daily variations in biochemical and physiological parameters between test and control groups.

Parameter	Arm	Day 1 (Baseline)	Day 2	Day 3	Day 4	Day 5	*p* Value (Total)
HCO3, median (IQR)	Test	22.0 (19.0–23.0)	23.0 (21.0–24.4)	20.7 (19.7–25.0)	22.8 (19.8–23.3)	24.6 (21.0–26.0)	0.141
	Control	23.5 (21.1–27.0)	25.0 (21.0–27.6)	25.7 (21.0–72.3)	23.0 (22.0–27.0)	24.3 (20.5–27.8)	0.214
	*p* value (subtotal)	0.341	0.011 *	0.002 *	0.124	0.714	
CO2	Test	36.0 (28.0–43.0)	43.0 (33.6–48.3)	37.5 (31.9–46.0)	40.0 (36.5–46.0)	43.1 (33.5–45.0)	0.332
	Control	36.7 (28.1–45.5)	31.6 (27.6–41.8)	34.1 (29.1–40.2)	40.0 (33.0–44.0)	42.2 (31.4–43.1)	0.147
	*p* value (subtotal)	0.481	0.021 *	0.078	-	0.147	
PH	Test	7.4 (7.2–7.5)	7.3 (7.2–7.4)	7.4 (7.3–7.5)	7.4 (7.3–7.5)	7.4 (7.3–7.5)	0.962
	Control	7.4 (7.3–7.5)	7.3 (7.3–7.6)	7.4 (7.3–7.5)	7.3 (7.2–7.4)	7.3 (7.2–7.6)	0.934
	*p* value (subtotal)	-	-	-	-	-	
Urea	Test	49.0 (42.0–170.0)	55.0 (50.0–218.0)	60.0 (52.0–170.0)	60.0 (54.0–150.0)	45.0 (44.0–145.0)	0.024 *
	Control	42.0 (28.0–93.0)	70.0 (50.0–212.0)	58.0 (33.0–143.0)	50.0 (40.0–150.0)	49.0 (35.0–144.0)	0.014 *
	*p* value (subtotal)	0.014	0.023	0.141	0.084	0.063	
Creatinine	Test	1.5 (1.1–2.7)	1.2 (1.1–2.8)	1.3 (0.9–1.4)	1.1 (0.9–1.4)	1.1 (0.9–1.3)	0.147
	Control	1.5 (0.9–2.7)	1.2 (1.1–3.2)	1.4 (0.8–3.2)	1.3 (0.8–3.4)	1.3 (0.9–3.2)	0.311
	*p* value (subtotal)	-	-	0.671	0.558	0.551	
Oxygen saturation	Test	92.0 (93.0–94.0)	96.0 (95.0–97.0)	95.0 (94.0–97.0)	95.0 (93.0–97.0)	94.0 (92.0–97.0)	0.557
	Control	94.0 (93.0–95.0)	95.0 (94.0–97.0)	95.0 (94.0–97.0)	93.0 (93.0–97.0)	93.0 (92.0–95.5)	0.478
	*p* value (subtotal)	0.147	0.127	-	0.214	0.741	
Temperature (Celsius)	Test	37.3 (37.0–37.4)	37.8 (37.4–37.9)	37.4 (37.0–37.6)	37.3 (37.0–37.4)	37.3 (36.9–37.5)	0.882
	Control	37.2 (37.1–37.5)	37.8 (37.2–37.9)	37.6 (37.0–38.5)	37.4 (37.2–37.5)	37.3 (36.9–37.5)	0.614
	*p* value (subtotal)	0.921	-	0.727	0.914	-	
Heart rate	Test	95.0 (82.0–104.0)	95.0 (84.0–96.0)	95.0 (73.0–96.5)	79.0 (73.0–87.0)	99.0 (86–99.0)	0.144
	Control	85.0 (82.0–97.0)	90.0 (74.0–95.0)	81.5 (73.0–92.0)	79.0 (69.0–92.0)	98.0 (84.2–99.5)	0.021 *
	*p* value (subtotal)	0.014 *	0.032 *	0.041 *	-	0.231 *	
Respiratory rate	Test	21.0 (21–25.0)	23.0 (20.0–27.0)	20.0 (19.0–20.0)	19.0 (18.0–22.0)	23.0 (20.0–31.0)	0.211
	Control	22.0 (20.0–25.0)	21.0 (17.8–22.3)	20.0 (19.0–26.0)	20.0 (17.0–22.0)	23.0 (20.0–31.0)	0.177
	*p* value (subtotal)	0.812	0.224	-	0.144	-	
WBC	Test	8.6 (8.6–16.0)	12.2 (11.4–16.4)	17.4 (10.1–17.4)	20.2 (11.8–22.0)	17.7 (12.2–18.0)	0.002 *
	Control	12.9 (8.6–16.075)	15.2 (12.2–17.5)	17.4 (12.0–18.4)	20.2 (11.0–22.0)	17.7 (10.5–18.0)	0.041 *
	*p* value (subtotal)	0.011 *	0.089	-	-	-	
Hemoglobin	Test	10.7 (10.6–11.6)	10.9 (9.9–11.4)	10.0 (9.8–11.1)	9.9 (9.5–11.0)	9.5 (9.5–10.5)	0.321
	Control	11.0 (10.6–11.4)	10.9 (9.4–12.8)	10.3 (9.7–11.5)	9.9 (9.5–11.2)	9.6 (9.0–10.5)	0.526
	*p* value (subtotal)	0.214	-	0.744	-	0.662	
Platelet	Test	231.0 (231.0–274.0)	255 (234.0–267.0)	244.0 (206.0–270.8)	244.0 (193.0 (273.0)	234.0 (190.0–261.0)	0.236
	Control	252.5 (231.0–274.0)	255 (224.0–270.0)	244.0 (198.7–271.5)	244.0 (193.0–296.0)	234.0 (182.0–298.0)	0.114
	*p* value (subtotal)	0.068	0.221	0.177	0.089	0.177	
Sodium	Test	137.0 (135.0–140.0)	141.0 (135.0–145.0)	142.0 (135.0–142.0)	139.0 (135.0–139.0)	142.0 (134.0–142.0)	0.811
	Control	135.0 (131.0–137.9)	135.6 (134.5–141.1)	137.0 (135.2–144.0)	136.0 (134.0–144.0)	135.0 (134.0–142.0)	0.905
	*p* value (subtotal)	0.144	0.205	0.144	0.441	0.277	
Potassium	Test	4.1 (4.0–5.0)	4.1 (4.1–5.1)	4.2 (4.1–4.9)	4.1 (3.8–4.6)	4.2 (4.0–4.3)	0.741
	Control	4.4 (2.8–4.8)	4.2 (3.0–4.9)	4.1 (3.0–4.4)	3.8 (3.3–4.1)	4.0 (3.0–4.3)	0.558
	*p* value (subtotal)	0.141	0.741	0.746	0.655	0.447	
ALT	Test	22.0 (12.0–22.0)	14.0 (14.0–17.0)	16.0 (16.0–26.0)	18.0 (18.0–33.0)	17.0 (17.0–37.0)	0.031 *
	Control	37.0 (22.0–44.0)	16.0 (14.0–46.0)	27.0 (18.0–66.0)	19.0 (18.0–42.0)	22.0 (15.0–43.2)	0.002 *
	*p* value (subtotal)	0.021 *	0.247	0.001 *	0.114	0.114	
AST	Test	31.0 (31.0–36.0)	20.0 (17.0–31.0)	23.0 (23.0–29.0)	22.0 (20.0–22.0)	22.0 (18.0–33.0)	0.003 *
	Control	35.0 (29.0–38.0)	31.0 (17.0–44.0)	27.0 (23.0–166.0)	25.0 (20.0–115.0)	29.0 (22.0–54.0)	0.095
	*p* value (subtotal)	0.114	0.021 *	0.074	0.089	0.001 *	
GLU	Test	164.0 (157.7–223.0)	170.0 (160.0–235.0)	205.0 (167.0–210.0)	160.0 (138.0–273.0(	207.0 (143.0–207.0)	0.001 *
	Control	163.0 (136.0–205.0)	163.0 (140.0–235.0)	205.0 (167.0–223.0)	165.0 (138.0–257.0)	187.0 (141.0–207.0)	0.126
	*p* value (subtotal)	0.741	0.556	-	0.114	0.001 *	

IQR: Interquartile range, WBCs: White blood cells, ALT: Alanine aminotransferase, AST: Aspartate aminotransferase, GLU: Glucose. * Statistically significant result *p* < 0.05.

**Table 5:** Change in mortality rate and ARDS development across the test and control groups.

		TEST	CONTROL
MORTALITY			
	Day 7	3 (4.8%)	4 (7.1%)
	Day 14	3 (4.8%)	4 (7.1%)
	Day 28	0 (0.0)	0 (0.0%)
	*p* value	0.212	0.122
ARDS DEVELOPMENT			
	Yes	6 (9.5%)	26 (46.4%)
	No	57 (90.5%)	30 (53.6%)
	*p* value	0.001 *	
NEED VENTILATION			
	Yes	6 (9.5%)	20 (35.7%)
	No	57 (90.5%)	36 (64.3%)
	*p* value	0.001 *	

* Statistically significant result *p* < 0.05. ARDS: Acute respiratory distress syndrome.

**Table 6:** Multiple linear regression analysis of factors influencing percent change in (S/F).

		Univariate Regression				Multivariate Regression		
Parameter	B Coefficient	SE	*95% CI*	*p*-Value	B Coefficient	SE	*95% CI*	*p*-Value
Type of Intervention	35.980	10.72	(14.95, 57.01)	0.001 *	46.406	12.610	(21.71, 71.10)	<0.001 *
Age	0.386	0.498	(−0.59, 1.36)	0.439	.709	0.503	(−0.28, 1.70)	0.162
Gender	16.339	11.405	(−6.03, 38.71)	0.155	14.091	12.592	(−10.60, 38.78)	0.266
Hemorrhagic stroke	−1.350	22.075	(−44.61, 42.26)	0.951	29.029	27.673	(−25.22, 83.28)	0.297
Pneumonia	2.645	11.302	(−19.51, 24.80)	0.815	7.377	13.310	(−18.70, 33.45)	0.581
Aspiration at admission	−26.762	11.144	(−48.61, −4.92)	0.018 *	−52.833	23.594	(−99.09, −6.58)	0.028 *
Smoking status	24.581	23.376	(−21.23, 69.39)	0.295	48.148	30.968	(−12.55, 108.85)	0.123
Aspiration **	−11.116	11.188	(−33.05, 10.82)	0.323	3.991	22.333	(−39.78, 47.77)	0.859
Shock **	−3.568	17.173	(−37.25, 30.12)	0.836	−5.179	21.597	(−47.51, 37.15)	0.811
Stroke **	9.742	11.388	(−12.58, 32.06)	0.394	24.209	14.157	(−3.54, 51.00)	0.091
Past admission ***	−16.324	19.873	(−55.27, 38.95)	0.413	−32.784	22.499	(−76.88, 11.31)	0.149
Ischemic heart disease	−28.960	14.875	(−58.13, 0.21)	0.054	−28.396	16.324	(−60.37, 3.97)	0.085
SOFA score	6.106	4.664	(−3.03, 15.25)	0.193	8.189	5.005	(−1.62, 17.00)	0.105
LIPS score	−1.047	3.631	(−8.17, 6.08)	0.774	0.328	3.946	(−7.41, 8.07)	0.934
ABG day 1	−10.856	12.163	(−34.68, 12.96)	0.374	−9.085	13.718	(−35.98, 17.81)	0.509
ABG day 2	0.699	14.537	(−27.81, 29.21)	0.962	0.244	19.176	(−37.09, 37.58)	0.990
ABG day 3	−7.757	12.912	(−33.09, 17.58)	0.549	−30.130	15.523	(−60.15, −0.11)	0.055
ABG day 4	6.819	11.266	(−15.26, 28.90)	0.546	18.649	13.269	(−6.61, 43.91)	0.163
ABG day 5	−7.777	11.641	(−30.61, 15.06)	0.505	−8.027	11.513	(−30.58, 14.53)	0.487

* Statistically significant result *p* < 0.05. ** Risk factors. *** Past admission within the last 90 days. Percent change of (S/F) is used as the independent variable. SOFA: Sequential Organ Failure Assessment, LIPS: Lung Injury Prediction Score, ABG: Arterial blood gas.

**Table 7:** Logistic regression analysis of factors associated with the development of ARDS.

Parameter	B Coefficient	Odds Ratio	95% CI (Lower, Upper)	*p* Value
Type of Intervention	−2.801	0.061	(0.01, 0.32)	<0.001 *
Age	−0.007	0.993	(0.95, 1.04)	0.748
Gender	−0.573	0.564	(0.18, 1.77)	0.312
Septic shock	1.304	3.683	(0.65, 20.87)	0.340
Pneumonia	0.629	1.876	(0.45, 7.86)	0.385
Aspiration	0.811	2.249	(0.48, 10.47)	0.299
Shock	−0.159	0.853	(0.16, 4.44)	0.852
Stroke	−0.277	0.758	(0.20, 2.91)	0.681
Past admission	1.356	3.879	(0.57, 26.70)	0.169
LIPS score	−0.159	0.853	(0.59, 1.23)	0.405
ABG day 1	0.102	1.108	(0.29, 4.22)	0.878
ABG day 2	0.349	1.418	(0.29, 6.96)	0.669
ABG day 3	1.122	3.070	(0.78, 12.05)	0.108
ABG day 4	−0.179	0.836	(0.23, 3.05)	0.783
ABG day 5	1.009	2.741	(1.00, 7.75)	0.057

Acute respiratory distress syndrome development is used as the independent variable. * Statistically significant result *p* < 0.05. LIPS: Lung Injury Prediction Score, ABG: Arterial blood gas.

**Table 8:** Logistic regression analysis of factors associated with the requirement for mechanical ventilation.

Parameter	B Coefficient	Odds Ratio	95% CI (Lower, Upper)	*p* Value
Type of Intervention	−1.960	0.141	(0.03, 0.63)	0.01 *
Age	0.027	1.027	(0.97, 1.08)	0.328
Gender	−0.746	0.474	(0.13, 1.66)	0.246
S/F Ratio (Day 5)	−0.004	0.996	(0.99, 1.00)	0.217
S/F Ratio percent change	−0.002	0.998	(0.97, 1.02)	0.874
Septic shock	1.471	4.353	(0.26, 71.45)	0.303
Pneumonia	0.686	1.985	(0.32, 12.39)	0.468
Aspiration	−0.282	0.754	(0.13, 4.22)	0.746
Shock	0.079	1.082	(0.16, 7.53)	0.938
Stroke	0.256	1.292	(0.32, 5.22)	0.722
Past admission	1.197	3.311	(0.45, 24.03)	0.237
LIPS score	−0.084	0.920	(0.62, 1.36)	0.680
ABG day 1	−0.299	0.741	(0.17, 3.26)	0.687
ABG day 2	1.702	5.484	(0.80, 37.33)	0.085
ABG day 3	0.067	1.069	(0.21, 5.49)	0.933
ABG day 4	−1.285	0.277	(0.06, 1.31)	0.114
ABG day 5	−0.044	0.957	(0.33, 2.80)	0.939

The need for mechanical ventilation is used as the independent variable. * Statistically significant result *p* < 0.05. S/F Ratio: peripheral oxygen saturation (SpO_2_)/fraction of inspired oxygen (FiO_2_) Ratio, LIPS: Lung Injury Prediction Score, ABG: Arterial blood gas.

**Table 9:** Multilevel modeling of S/F ratio and treatment interactions.

Fixed Effects			Random Effects		
Parameter	F Value	*p* Value	Parameter	Covariance Estimate	S.E.
Intercept	95.691	<0.001 *	Day 1	8506.38	1175.70
Group	18.125	<0.001 *	Day 2	9719.71	1321.89
Time	1.751	0.141	Day 3	12,752.88	1735.59
Group × time	2.905	0.023 *	Day 4	26,407.38	3594.80
Group × pulmonary edema	7.071	<0.001 *	Day 5	27,300.61	3749.54
Group × pneumonia	17.660	<0.001 *	Gender	1049.09	1705.32
			Aspiration	539.97	1015.11
			Shock	198.93	767.16
			Ischemic heart disease	1876.99	3010.51
			LIPS score	46.38	87.58
			SOFA score	104.73	180.27

* Statistically significant result *p* < 0.05. S/F Ratio: peripheral oxygen saturation (SpO_2_)/fraction of inspired oxygen (FiO_2_) Ratio, LIPS: Lung Injury Prediction Score.

## Data Availability

Availability of Data and Materials: Data are available from the corresponding author (H.A.M.M.) upon reasonable request. The data are not publicly available due to confidentiality and patient privacy.

## Supplementary Materials

The following supporting information can be downloaded at: https://www.mdpi.com/article/10.3390/ph18030412/s1.
